# Simple and Feasible Detection of Hepatitis B Virus *via* Combination of Multienzyme Isothermal Rapid Amplification and Lateral Flow Dipstick Strip

**DOI:** 10.3389/fmolb.2021.763079

**Published:** 2021-12-02

**Authors:** Mao-Ling Sun, Hai-Yun Lai, Na-Yu Chong, Dong-Fan Liu, Zhen-Yi Zhang, Bo Pang, Jun Yao

**Affiliations:** School of Forensic Medicine, China Medical University, Shenyang, China

**Keywords:** hepatitis B virus, multienzyme isothermal rapid amplification, lateral flow strip, detection, nucleic acid

## Abstract

Hepatitis B virus infection is not only a huge burden in the field of social health but also a major public health problem that affects the lives and health of the people. Simple, rapid, feasible detection of HBV is critical for its prevention and spread, especially in the developing countries with low-resource laboratories. To this end, we combined multienzyme isothermal rapid amplification (MIRA) and lateral flow dipstick (LFD) strip to detect HBV. A pair of primers targeting the conserved region of HBV genome was designed and used in MIRA-LFD assay. Our results found that the entire amplification of MIRA-LFD only takes 10 min at 37°C and the dilution of the amplification products was added in the LFD strip and observed by the naked eye after 10 min. The detection sensitivity of this method can reach 10 pg. The 45 clinical samples were detected by MIRA-LFD and real-time PCR. The accuracy rate of MIRA-LFD was 100%. Therefore, these characteristics of our newly developed MIRA-LFD assay make it particularly useful and suitable for detecting HBV in the resource-limited condition.

## 1 Introduction

Hepatitis B virus (HBV) belongs to the hepatophilia deoxyribonucleic acid (DNA) viridae and can be divided into positive hepatophilia DNA virus and poultry hepatophilia DNA virus. In the former, HBV is one of the main pathogens of human liver disease. Currently, HBV includes nine genotypes (A∼I), as well as different subtypes of the respective genotypes, according to the phylogenetic analysis of whole genome sequence (Demosthenes et al., 2019). HBV can cause people to get hepatitis B. The main routes of its transmission include blood transmission, mother-to-child transmission, and medical-borne transmission (Li et al., 2021). The patients can appear with loss of appetite, nausea, upper abdominal discomfort, liver pain, fatigue, and other symptoms. After the HBV invades the human body, it gives rise to cellular immunity restriction, causing a series of continuous liver inflammatory reactions, resulting in the continuous aggravation of the immune injury of the body, which may eventually develop into cirrhosis and even into liver cancer (Nagaoki et al., 2021).

There are 70 million hepatitis B virus carriers in China, and every year, the number of new cases of hepatitis B is stable at about 1 million (Lu and Zhuang, 2009). The direct medical expenses for the treatment of hepatitis B and related liver diseases in China reach to 100 billion yuan every year. Furthermore, the blood transmission of HBV *via* contaminated blood products is of enormous threat in the case of emergency transfusions, especially in resource-limited regions, which are endemic for HBV. Therefore, hepatitis B is not only a burden in the field of social health economy but also a major public health problem that threatens the life and health of Chinese people. At present, the clinical application of anti-viral treatment can significantly improve the prognosis of patients. However, because the virus DNA in the nucleus cannot be completely removed, patients may have HBV reactivation phenomenon (Perrillo et al., 2015) and need to take medicine for life, and there is no effective radical cure. Therefore, an accurate, economical, and efficient pathogen diagnosis is the key to control the infection of HBV.

Presently, the detection method of HBV widely used in the clinic is fluorescence quantitative polymerase chain reaction (FQ-PCR), which has strict requirements to laboratory facilities and professionals. Thus, it is not suitable for popularization in a resource-limited area (Weber et al., 2019). Another method, enzyme-linked immunosorbent assay (ELISA) usually takes 1∼2 days to get the detection result, and the antibody preparation cycle is long (Ye et al., 2021).

In recent years, several isothermal technologies have been developed for rapid pathogen detection. Multienzyme isothermal rapid amplification (MIRA) technology, similar to recombinase polymerase amplification (RPA), is a novel rapid isothermal amplification technique of nucleic acid. This technology uses four core proteins, namely, recombinase-Rec A, DNA helicase-gp41, single-stranded binding (SSB) protein, and DNA polymerase-DNA pol I (Piepenburg et al., 2006). The helicase could cooperate with SSB to form the D-loop rapidly (Lu et al., 2021). The reaction can be completed within 5–30 min under the condition of 25°C–42°C, which can effectively avoid the disadvantages of the above isothermal amplification technology and is sensitive, specific, economic, and convenient. In addition, the lateral flow strip bands are easy to observe, and the results can be visually evaluated as positive or negative. This can also be done by the untrained personnel. Therefore, the interpretation of the lateral flow strip and the processing of the reaction itself require minimal equipment and no special trained operator (Liu et al., 2018; Miao et al., 2019). In addition, with the help of MIRA-LFD, the lateral flow strip can replace the other expensive equipment currently in use, to reduce the cost of each test. Thus, the detection of HBV by MIRA-LFD has the potential application in the developing countries with low-resource laboratories.

In order to solve the current problem of virus detection and promote the early detection and early treatment of hepatitis B in resource-limited rural area, our study combined MIRA and LFD technology to provide a rapid and easy to implement diagnosis method of HBV.

## 2 Methods

### 2.1 Primer Design

The primers were designed following the guidelines of AMP-Future Biotech Co. Ltd. (Weifang, China), which target the conserved region of the HBV genome. According to the guidelines, the length of primer was approximately 25–35 bp, whereas the amplicon was approximately 150–300 bp. A total of 17 HBV genome sequences were downloaded from NCBI and sequence comparison was performed using DNAMAN 9.0 software. The conserved X and S gene sequences were selected for primers design using Premier 5.0 software. The specificity analysis of the primers was screened by BLAST search (http://www.ncbi.nlm.nih.gov/blast/Blast.cgi).

### 2.2 Hepatitis B Virus Deoxyribonucleic Acid Preparation

The plasmid DNA with HBV fragment was used as template for MIRA reaction. A 3,418 bp DNA fragment from HBV genotype B was cloned into pCDNA3.1(+) by TSINGKE Biotech Co. Ltd. (Nanjing, China). After plasmid extraction, the DNA concentration was measured by NanoDrop™ One UV spectrophotometer (ThermoFisher Scientific, Waltham, MA, USA), and the copy number was calculated according to Yi et al.’s (2021) study.

### 2.3 Multienzyme Isothermal Rapid Amplification Basic Reaction

The MIRA basic reactions were performed using the kit (# WLB8201KIT) from AMP-Future Biotech Co. Ltd. According to the instruction of the manufacturer, the reaction was performed in a 39°C water bath for 30 min. After purified and diluted at 1:10,000, a 10-μl product was detected in 2% agarose gel electrophoresis. After electrophoresis at 110 V for 50 min, the gel was visualized by automatic digital gel image analysis system (Tanon, Shanghai, China).

### 2.4 Multienzyme Isothermal Rapid Amplification-Lateral Flow Dipstick Reaction

For the MIRA-LFD reactions, the forward and reverse primers were labeled with FAM and Biotin at the 5′-end, respectively. The reactions were performed using the kit (# WLB8203KIT) from AMP-Future Biotech Co. Ltd. According to the instruction of the manufacturer, the reaction was performed in a 37°C water bath for 10 min, and the product was diluted with H_2_O at the ratio of 1:5,000. Then a 100-μl diluent was added in the lateral flow nucleic acid test note (# JY0201, Baoying Tonghui Biotechnology Co. Ltd., Beijing, China), and the color was observed after 10 min.

### 2.5 Sensitivity and Specificity Analysis

To determine the detection limit of the MIRA-LFD reaction, a serial dilution of the HBV plasmid DNA was amplified and measured in gel electrophoresis. Moreover, the HBV plasmid DNA diluted from 1–10 fg was amplified and detected in colloidal gold lateral flow dipstick detection. In addition, the template of human genome was used to identify the specificity of the MIRA-LFD reaction using colloidal gold lateral flow dipstick. Moreover, the HIV-1, HSV-1, JEV, HAV, and HCV nucleic acids were also selected to validate the cross-reactivity of the MIRA assay. In addition, a pair of qPCR primers was also added for the sensitivity comparison (Table 1) (Kania et al., 2014). The qPCR reaction was conducted using TaKaRa TB Green Premix Ex Taq II (Tli RNaseH Plus) (TaKaRa, Japan) in the Applied Biosystems 7500-Real Time PCR system (Thermo Fisher Scientific). Each reaction consisted of at 95°C for 30 s, followed by 40 cycles at 95°C for 5 s and 60°C for 34 s. All the amplification reactions were performed in triplicate.

**TABLE 1:** The primers used in quantitative polymerase chain reaction (qPCR) reaction.

Primer	Sequence (from 5′ to 3′)	Product length
HBV-TAQ1	GTG​TCT​GCG​GCG​TTT​TAT​CA	98 p
HBV-TAQ2	GAC​AAA​CGG​GCA​ACA​TAC​CTT	

### 2.6 Hepatitis B Virus Detection From Clinical Samples

A total of 45 blood samples were collected from the local hospital. Among them, 26 samples were confirmed as HBV positive. The DNA of these samples was extracted by QIAamp Blood DNA Kit (Qiagen, Germany) according to the instruction of the manufacturer. Then the extracted DNA was amplified using MIRA-LED reaction and detected in colloidal gold lateral flow dipstick.

## 3. Results

### 3.1 The Selection of Primer

**FIGURE 1:**
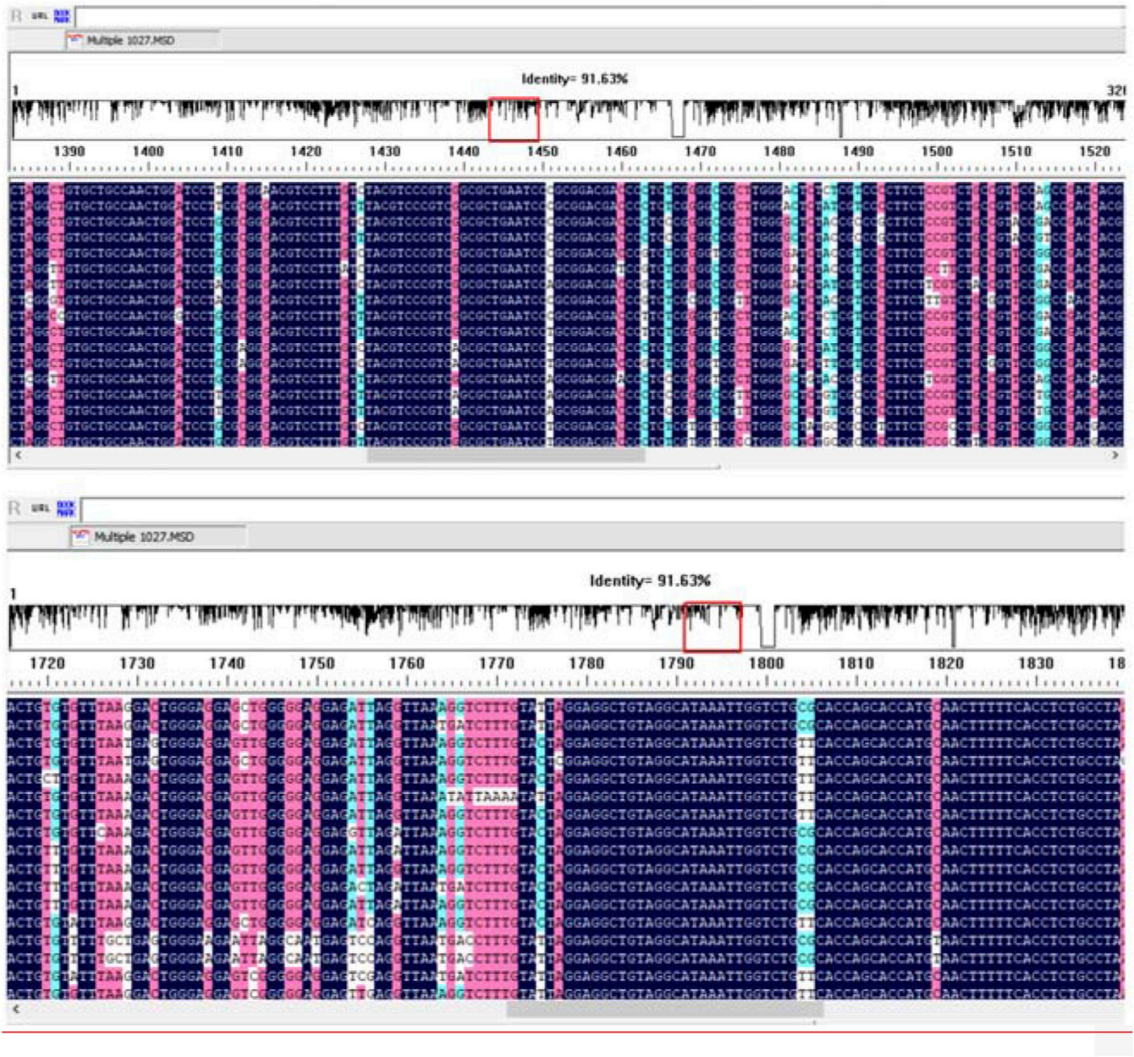
The alignment of 17 hepatitis B virus (HBV) genome sequences using the DNAMAN software.

The DNAMAN software was used to blast the sequence of HBV subtypes. However, no appropriate conserved sequence could be selected for primer design (Figure 1). We mainly selected the HBV subtypes B and C, which were epidemic in East Asia and China, for primer design, since our study was mainly planned to be applied in China.

**FIGURE 2:**
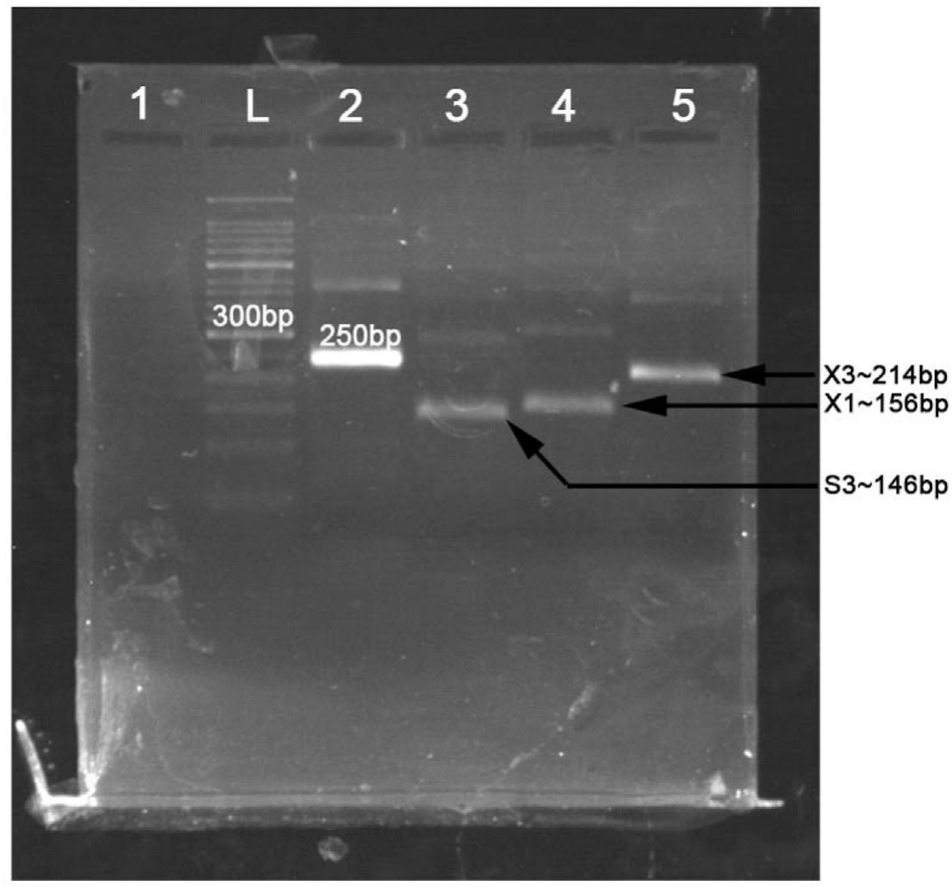
The products of multienzyme isothermal rapid amplification (MIRA) reaction measured in 2% agarose gel electrophoresis. L, 50-bp DNA ladder; 1, negative control; 2, positive control (∼250 bp); 3, product of primer S3 (∼146 bp); 4, product of primer X1 (∼156 bp); 5, product of primer X3 (∼214 bp).

The primers used in MIRA reaction are shown in Table 2. The MIRA basic reaction was performed by the unlabeled primers at 39°C for 30 min to test the usability of the primers. The result showed that all the three target fragments can be successfully amplified (Figure 2). Among them, primer X3 exhibited the best efficiency.

**TABLE 2:** The primers used in multienzyme isothermal rapid amplification (MIRA) reaction.

Primer	Sequence (from 5′ to 3′)	Target	Product length (bp)
S3-F	CAG​GAA​CAT​CAA​CTA​CCA​GCA​CGG​GAC​C	S gene	146
S3-R	TGC​GAA​CCA​CTG​AAC​AAA​TGG​CAC​TA	S gene	
X1-F	CGG​TCT​CCC​CGT​CTG​TGC​CTT​CTC​ATC​TGC	X gene	156
X1-R	AGG​TCG​GTC​GTT​GAC​ATT​GCT​GAG​AGT​CCA​AGA​GT	X gene	
X3-F	TAC​CGT​CCC​CTT​CTC​CGT​CTG​CCG​TTC​CG	X gene	214
X3-R	AGG​TCG​GTC​GTT​GAC​ATT​GCT​GAG​AGT​CCA​AGA​GT	X gene	
X3-F (labeled)	[5′-FAM]-TACCGTCCCCTTCTCCGTCTGCCGTTCCG	X gene	214
X3-R (labeled)	[5′-biotin]-AGGTCGGTCGTTGACATTGCTGAGAGTCCAAGAGT	X gene	

### 3.2 The Sensitivity and Specificity of Multienzyme Isothermal Rapid Amplification Basic Reaction

**FIGURE 3:**
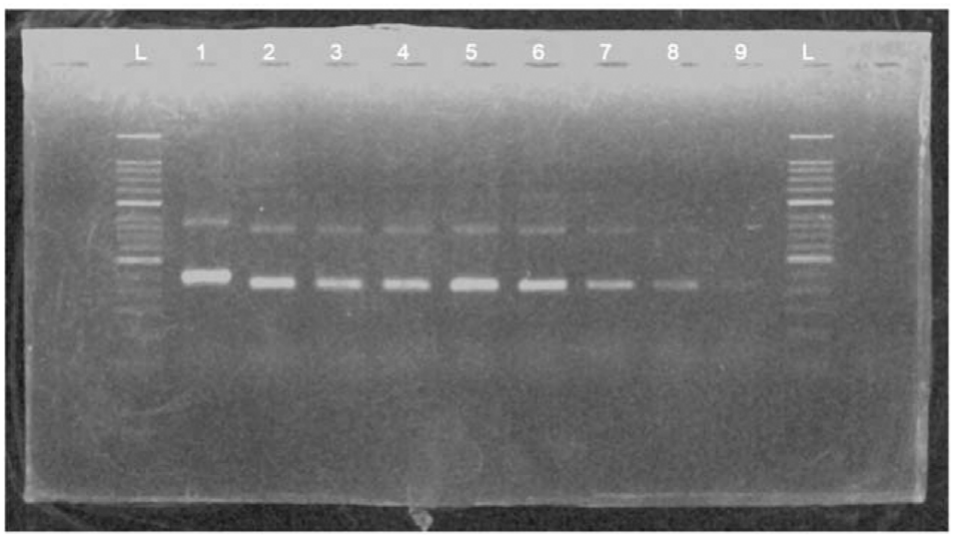
The products of a serial dilution of HBV plasmid deoxyribonucleic acid (DNA) measured in 2% agarose gel electrophoresis. L, 50 bp DNA ladder; 1, positive control (∼250 bp); 2, product of 25-ng template; 3, product of 5-ng template; 4, product of 1-ng template; 5, product of 200-pg template; 6, product of 100-pg template; 7, product of 50-pg template; 8, product of 25-pg template; 9, product of 12.5-pg template.

To determine the detection limit of the MIRA basic reaction, a serial dilution of HBV plasmid DNA was amplified using primer X3 in MIRA reaction and detected in 2% agarose gel (Figure 3). The results found that the templates from 25 ng to 25 pg could be detected successfully in agarose gel. However, the template of 12.5 pg could not be observed clearly.

**FIGURE 4:**
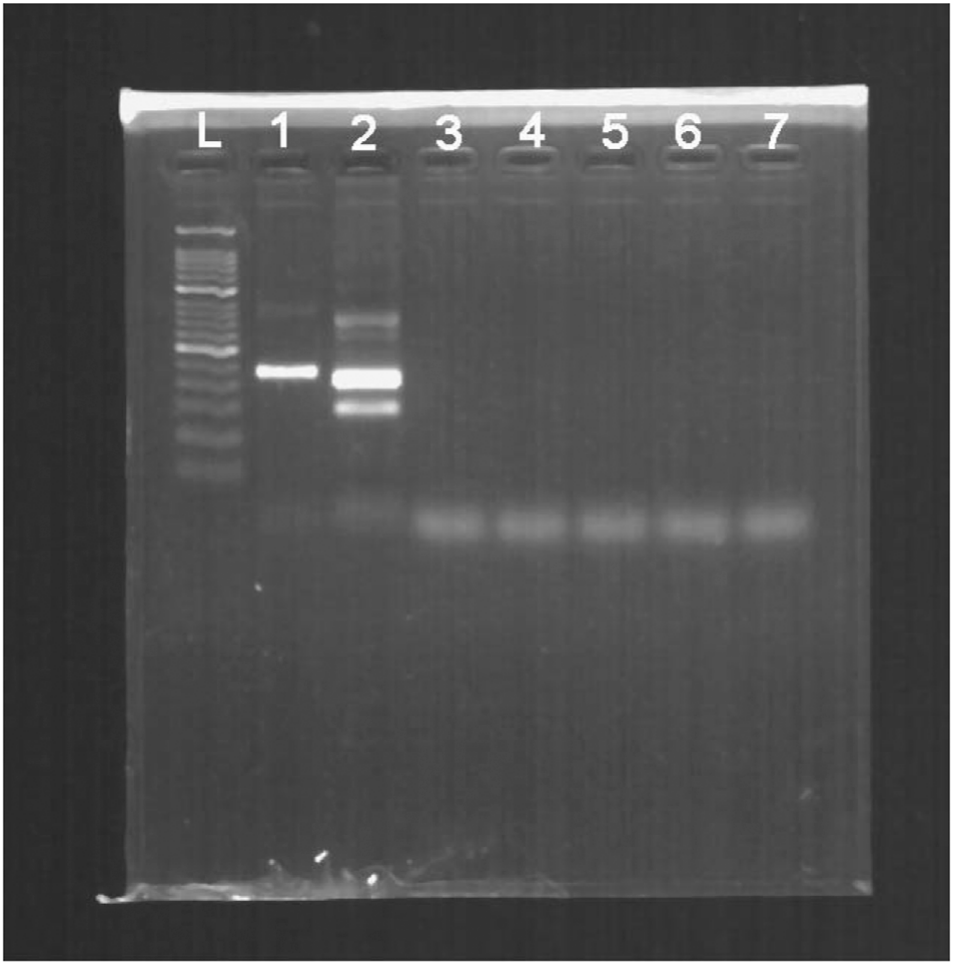
The cross-reactivity test of multiple pathogens measured in 2% agarose gel electrophoresis. L, 50-bp DNA Ladder; 1, positive control (∼250 bp); 2, product of HBV; 3, product of HIV-1; 4, product of HSV-1; 5, product of JEV; 6, product of HAV; 7, product of HCV.

Additionally, no positive results were observed in the templates from the other pathogens expect for HBV (Figure 4).

### 3.3 Multienzyme Isothermal Rapid Amplification-Lateral Flow Dipstick Reaction

**FIGURE 5:**
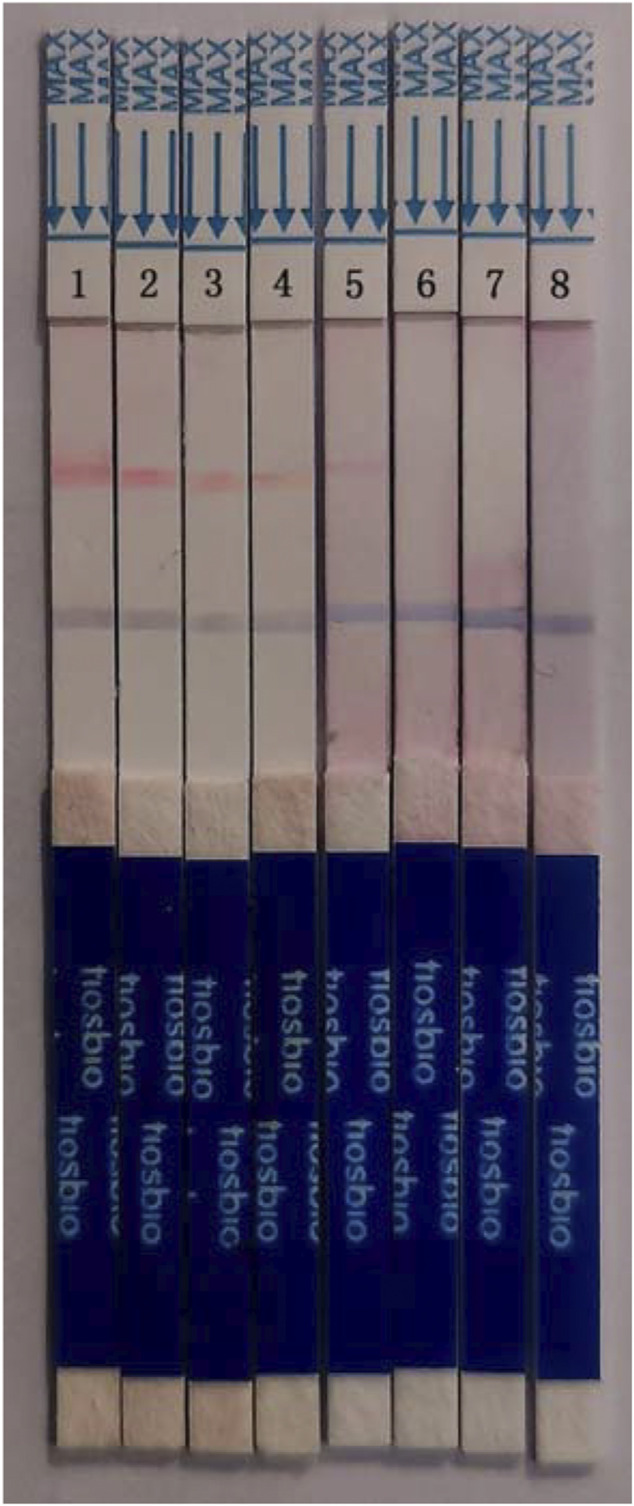
The products of a serial dilution of HBV plasmid DNA measured in MIRA-LFD. 1, product of 1 ng template; 2, product of 100 pg template; 3, product of 10 pg template; 4, product of 1 pg template; 5, product of 100 fg template; 6, product of 10 fg template; 7, product of human genome template; 8, product of water template.

**FIGURE 6:**
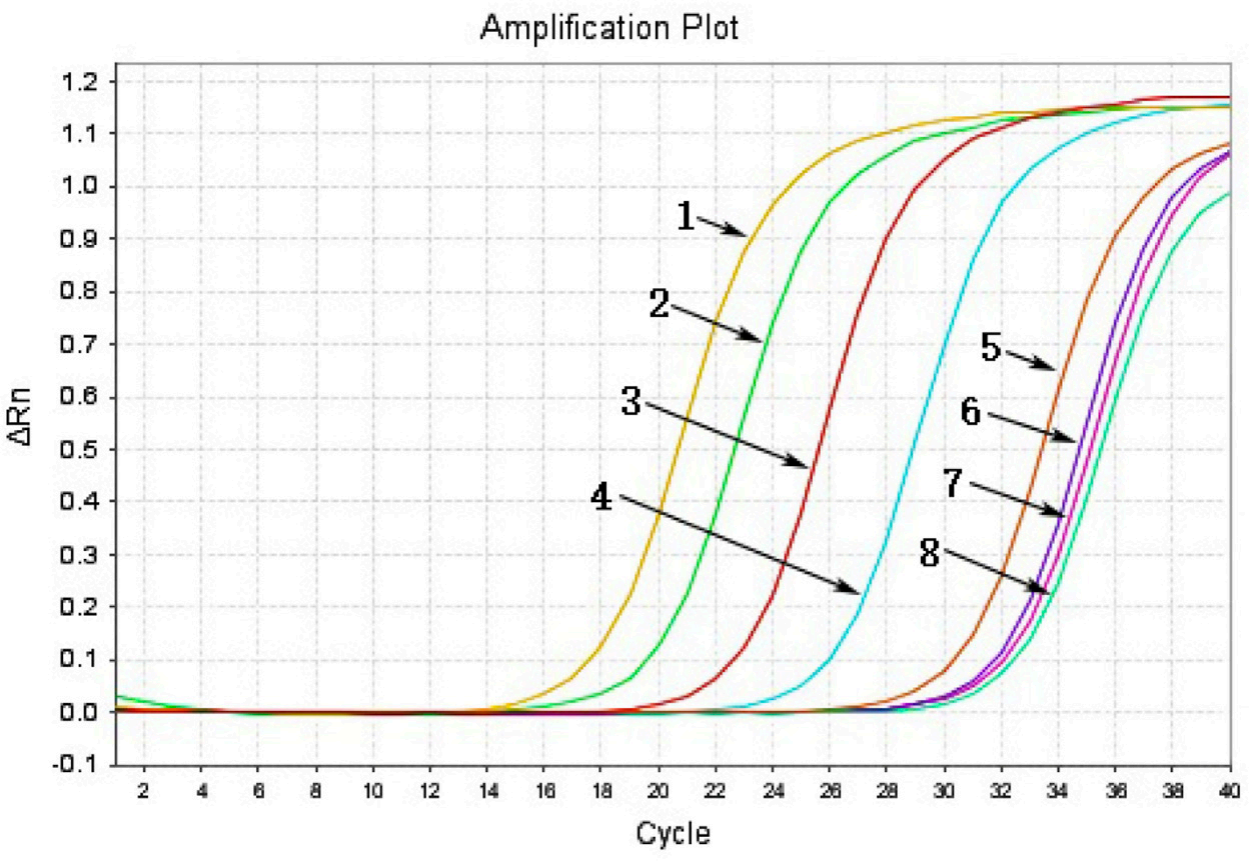
The products of a serial dilution of HBV plasmid DNA measured in qPCR. 1, product of 100-pg template; 2, product of 10-pg template; 3, product of 1-pg template; 4, product of 100-fg template; 5, product of 10-fg template; 6, product of 1-fg template; 7, product of 0.1-fg template; 8, product of water template.

To identify the sensitivity of the MIRA-LFD reaction, the HBV plasmid DNA was diluted into 1 ng, 100 pg, 10 pg, 1 pg, 100 fg, and 10 fg. Then six dilutions were amplified and detected in colloidal gold LFD (Figure 5). The results showed that even the 100-fg plasmid DNA could be detected in LFD, while the template of water and human genomic DNA showed a negative result. In addition, the different dilutions of HBV genome template from 100 pg to 0.1 fg were detected by qPCR assay, and the detection limitation was found to be 100 fg (Figure 6). The results showed that MIRA-LFD have the similar sensitivity as qPCR.

### 3.4 The Detection of Clinical Samples

The MIRA-LFD and real-time PCR were used to test the HBV in 45 clinical samples. All the 26 positive samples and 19 negative samples were accurately detected (Table 3). The 100% accuracy rate in the results of clinical sample suggested that MIRA-LFD is suitable for HBV detection in clinical application.

**TABLE 3:** The results of hepatitis B virus (HBV) detection in the clinical samples.

		Real time-PCR			Accuracy rate
		positive	negative	total	
MIRA-LFD	positive	26	0	26	100%
	negative	0	19	19	
	total	26	0	45	

## 4 Discussion

In this study, we designed specific primers in two highly conserved regions (region S and X) of the HBV genome and established an HBV DNA detection method *via* MIRA-LFD reaction. The entire amplification of MIRA-LFD only takes 10 min at 37°C, and then the dilution was added in the test trip and observed by the naked eye after 10 min. Compared with the existing method, this method is efficient and suitable to implement in the resource-limited area without the restriction of laboratory condition. In the MIRA-LFD reaction, the detection sensitivity can even reach 10 pg. Therefore, it is helpful to detect the potential HBV infection (Tu et al., 2017) and has the significant application in the prevention of the spread and infection of HBV.

With the rapid advancement of molecular biotechnology, several isothermal amplification techniques have been developed. Multiple cross displacement amplification (MCDA) (Wang et al., 2015), loop-mediated isothermal amplification (LAMP) (Nagamine et al., 2001; Wong et al., 2018), and cross-priming amplification (CPA) (Fang et al., 2009) can be carried out at about 60°C–65°C, and the amplification process usually ends within 60 min. In addition, the process of primer design is very complicated, and more than four primers need to be designed for amplification. Isothermal polymerase spiral reaction (PSR) is also performed at about 60°C for 60 min, and only one pair of primers need to be designed (Liu et al., 2015). Compared with the abovementioned methods, the RPA is mild and efficient. The reaction is performed at 37°C–42°C for 20–40 min and only needs to design one pair of primers (Piepenburg et al., 2006). However, it has been reported that RPA is not ideal for discriminating between mutations or identifying mutations based on the performance of a nested RPA-based assay (Ng et al., 2017).

**FIGURE 7:**
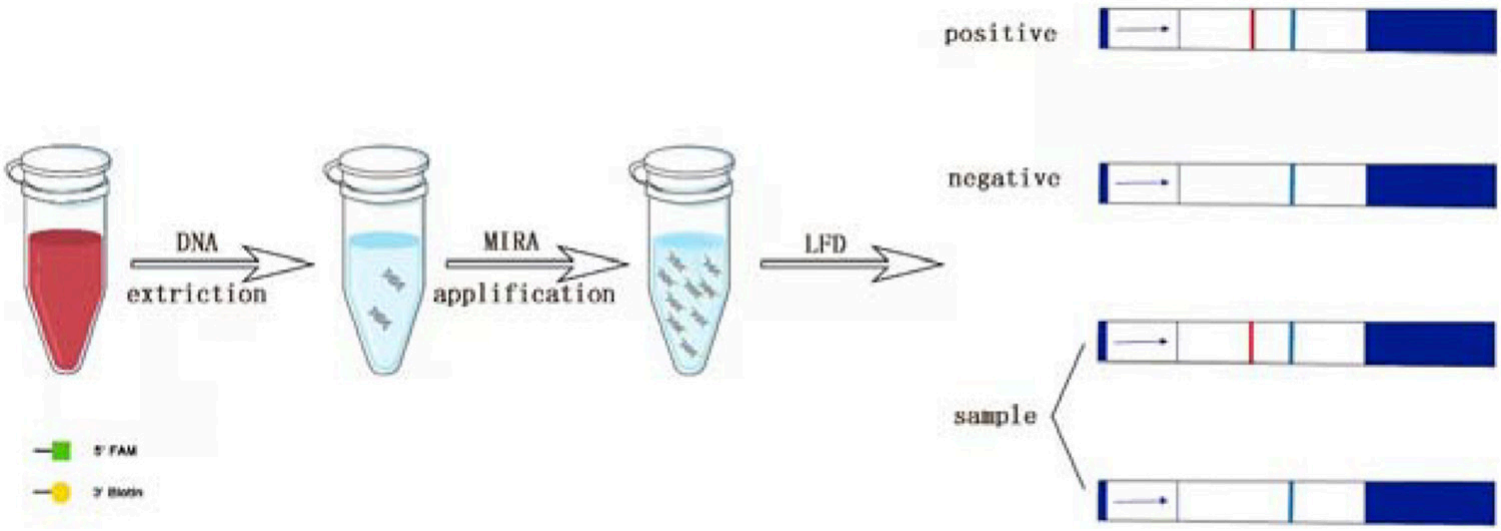
Scheme summarizing the entire process.

The MIRA assay can be reacted continuously at 37°C–42°C with a reaction time of only 5–30 min. Additionally, it does not need the strict laboratory condition (Piepenburg et al., 2006). Therefore, it is appropriate in initial screening of infectious diseases. Lateral flow dipstick (LFD) technology is a high-sensitivity technique for the detection of trace antigens, which uses an amplified product containing a biotin to hybridize with the probe labeled 5-carboxyfluorescein (FAM) and bind to colloidal gold-labeled antibodies, to obtain intuitive detection results in a short time. Now, combined with other isothermal technologies, it has exhibited a great potential in rapid pathogen detection (Zeng et al., 2019) (Figure 7).

There are still several potential limitations in the present study. First, due to the numerous subtypes of hepatitis B virus, the primer sequence can only identify some of these subtypes, but not all of them. The HBV subtypes that do not match the primer-binding regions cannot be detected. To some extent, it limits the wide usage of this method. Second, DNA extraction process is still required. Although some reagent, such as chelex-100, has simplified the DNA extraction process, the DNA extraction process is still a limitation of the entire process. Our method did not combine the DNA extraction and rapid amplification into one step. Third, the present method contains only one MIRA reaction and one LFD strip, which is difficult to detect the HBV with mutations in the primer recognition sequence. It is possible to add additional reaction and test strip for detecting other mutations in the future optimization of MIRA-LFD.

## 5 Conclusion

In this study, the combination of MIRA and LFD is a simple and feasible method without the restriction of special instrument. The entire detection of MIRA-LFD only takes 10 min at 37°C, and then the dilution was added in the test trip and observed by the naked eye after 10 min. This fast and easy-to-read-out method has the potential application in the developing countries with low-resource laboratories.

## Data Availability

The original contributions presented in the study are included in the article/Supplementary material. Further inquiries can be directed to the corresponding author.
